# Research of Vibrations of an Armoured Personnel Carrier Hull with FE Implementation

**DOI:** 10.3390/ma14226807

**Published:** 2021-11-11

**Authors:** Zdzisław Hryciów, Jerzy Małachowski, Piotr Rybak, Andrzej Wiśniewski

**Affiliations:** Faculty of Mechanical Engineering, Military University of Technology, gen. Sylwestra Kaliskiego Street 2, 00-908 Warsaw, Poland; zdzislaw.hryciow@wat.edu.pl (Z.H.); jerzy.malachowski@wat.edu.pl (J.M.); piotr.rybak@wat.edu.pl (P.R.)

**Keywords:** military vehicle, modal analysis, roving hammer, vibration

## Abstract

Modern wheeled armoured vehicles can perform a variety of tasks, making the development of weapon systems that can be safely and effectively integrated with the vehicle structure an area of interest. Due to the cost of implementing new models, it is more economical to test potential configurations using numerical methods, such as the finite element method. The numerical model has been validated to confirm the reliability of the obtained results. Modal tests were also performed using four configurations to identify the frequency and mode shape of natural vibrations occurring within the support structure. In an experimental setting, hull vibrations were forced using the modal hammer testing method. The modal assurance criterion (MAC) and the authors’ procedure were used to confirm the experimental and numerical test results. Additional testing in the form of impact loads was carried out for turret-containing structures. Structural strain at indicated points and forces transmitted by brackets to the bottom of the hull were compared.

## 1. Introduction

The effectiveness of military-related tasks performed by multi-axle armoured vehicles depends on the acceptance of the expected operating conditions at the design stage. This generally translates to the establishment of appropriate tactical, technical, and design requirements. Limit values must also be established for relevant parameters, describing characteristics such as firepower of the basic armament, ability to protect people and internal equipment, and the ability to drive in various road conditions. These features are also shaped based on the expected range of applications. An important factor influencing the geometry and overall design of the vehicle is the weapon system—including the mass, calibre, and recoil of the cannon. The design is intended to be modular. This approach provides a well-prepared support structure (e.g., self-supporting body and hull), capable of using special equipment that will generate shock loads of varying intensity. An important structural node in this case is the connection of the roof hull plate and the turret system. Armament systems for this vehicle class can generate loads in the full angular range in the horizontal plane and −10° to 60° in the vertical plane. Due to the fact that these kinds of vehicles should be as lightweight and small in size as possible, special bodies must be designed based on the turret system. Thus, the focus on universality of body structure on the one hand and weight minimisation on the other creates additional requirements that may oppose one another [1]. Therefore, when designing special bodies, we do not talk about their optimisation, but rather about the compromises taken. Thus, before deciding to change the purpose/equipment of an armoured vehicle, it is necessary to carry out appropriate testing.

A common practice used by R&D institutions during the design and development of new constructions is the numerical approach. There are two common approaches, one based on modelling of rigid bodies and the other relying on the finite element method (FEM). For example, in [2], a four-wheeled vehicle with a mounted mortar is modelled using a six-degrees-of-freedom model. The effects of firing the weapon (impact amplitude, duration, and elevation angle) on vehicle are investigated. However, FEM analysis gives much wider possibilities, such as modal analysis and evaluation of a structural response to static and dynamic loads. In [3,4], tests were carried out to determine crew and special equipment exposure to hazards such as direct fire, shrapnel, explosion of anti-tank mines, or improvised explosive devices. As a result, this approach allows for the development of solutions providing improved protection [5,6,7,8] and parameter optimisation [9] in order to minimise crew exposure as much as possible. Another important aspect to be included in the design process is compliance with international requirements, such as the NATO STANAG 4569.

Numerical methods have a wide range of applications, including simulating the effects of different protective equipment and armour configurations on the degree of protection for the crew, internal equipment, traction, and operational properties. The advantage of this method, when combined with relevant data and compared with real object testing, lies in a significant cost reduction, identification of sensitive structural nodes, and determination of resonant frequencies—all of which can have a major impact on the durability and reliability of specialised equipment as well as on weapon accuracy [10]. However, to ensure the reliability of the obtained results, validation of the numerical model must be performed. Many different approaches to this are presented in the literature. The most reliable method is to conduct full-scale experimental research on real objects. Unfortunately, this kind of testing is expensive and, in the case of armoured vehicles, often leads to the destruction of the test subject. An alternative approach is to conduct research using replacement models or isolated parts of the structure. However, this does not always yield correct predictions for the vehicle behaviour as a whole.

The validity of the finite element model for a particular structure is an important step in the solution process. The experimental modal analysis correlates with the finite element model through the evaluation of the structure dynamics. The modal properties (such as natural frequencies and mode shapes) are the parameters used to correlate the finite element results [11,12].

Valuable information about the dynamic properties of technical objects is provided through the modal analysis. This technique is used in many technological fields and validates the numerical model. This approach was also used previously [13,14] for combat vehicle hull applications, including the fragments of protective structures. In another study [15], the authors presented the results of the experimental and numerical modal analysis of the bottom of the hull of an armoured personnel carrier. To determine the frequency and mode shape of natural vibrations for comparison, both the standard approach using the mode indicator function (MIF) and the approach using the modal assurance criterion (MAC) indicator were used. The authors’ procedure for comparing the mode shape of vibrations using an interpolation function was also presented. Structure excitation was carried out by using an impact hammer. This method allows for study of structures with both small and large dimensions, regardless of the construction materials. Other literature reports [16] presented results for composite plate modal studies, along with the possible applications for validating using numerical models. For very large and complex structures, a special approach should be taken to determine dynamic properties. Due to the lack of possible external excitation, the natural vibration generation of the working device was used for analysis. An example of this type of approach was presented in the literature [17] for a surface mining machinery where an experimental application was used to determine the modal characteristics and subsequently upgrade the working elements. Similar considerations were also presented in other works [18], where experimental results were compared with numerical results. 

In the literature there are papers pointing out to a significant influence of vibrations of weapon system structural components on the firing accuracy [10,19,20]. However, the presented results are limited to barrel vibrations. In [19] examples of considerations related to the vibrations of a tank cannon were presented, including the influence of barrel vibrations of a 120 mm cannon on projectile behaviour. An experimental–numerical analysis of machine gun barrel vibrations during active firing has also been investigated [20]. Pressure changes within the barrel and their impact on dynamic weapon structure loading were also considered. In weapon systems characterised by high firing rates, the weapon attachment method becomes extremely important. The generated high-frequency recoil force may cause structural vibrations that reduce accuracy. Reports in the literature discussed vibration reduction problems along with improved firing accuracy for a machine gun mounted on a tripod base [21]. The strong influence of the rifle’s self-supporting structure vibrational frequency on the firing accuracy was demonstrated, as well as optimisation possibilities for dynamic structure properties.

The main objective of this work was to verify the accuracy of the numerical model that simulates the hull of an armoured personnel carrier using results from a modal test performed on the basis of the modal hammer method. The comparison was completed using standard tools (i.e., MIF function and MAC criterion). The uniqueness of the work lies in the application of an original procedure to compare the form of natural vibrations obtained from experimental and model tests. The frequencies and modes of natural vibrations of the hull plate of the vehicle and the influence of the introduced equipment on their values, obtained by means of experiments, are also an original result of the work. The novelty of this work involves the application of an original procedure to compare the mode shape of natural vibration obtained from experimental and FE tests. The unique results of the study also include the natural frequencies and mode shape of vibrations of the vehicle hull plate (obtained experimentally) and the influence of the introduced equipment elements on their values.

## 2. Methods

### 2.1. Experimental Study

**Figure 1:**
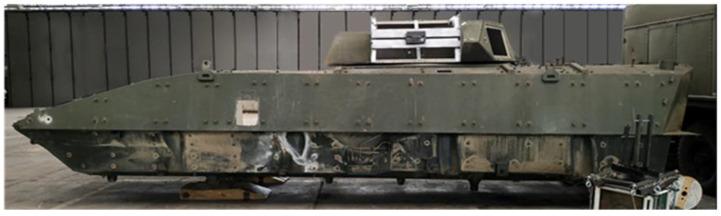
Test object view.

Modal analysis was used to identify important object properties and to determine the natural vibration frequency and corresponding vibration mode shapes. The damping factors of objects with complex structures could also be evaluated. A view of the test subject, the hull of an armoured personnel carrier, is shown in Figure 1.

**Figure 2:**
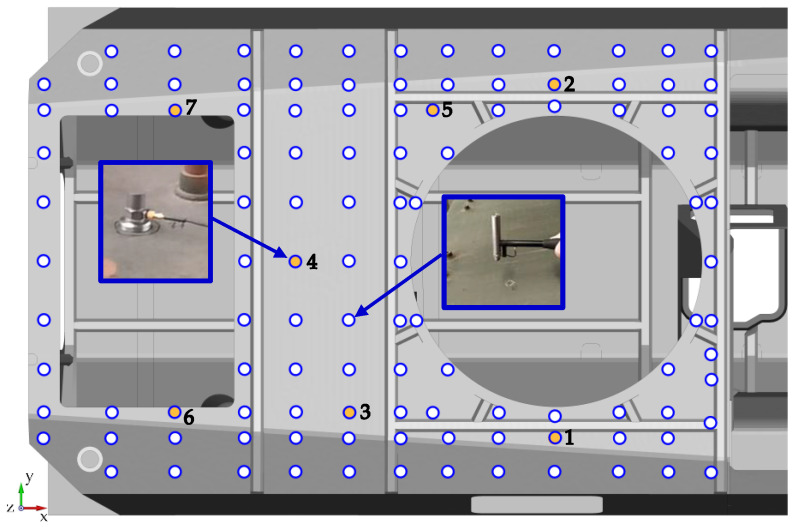
Measuring point grid and location of accelerometers (white and orange dots).

This study was carried out using experimental modal analysis, a technique commonly used for studying dynamic mechanical object properties, at both the design and machine operation stages. Identification tests used for experimental modal analysis rely on forcing an object to vibrate while simultaneously measuring the driving force and system response, often in the form of vibrational acceleration spectra. The tests used a B&K type 8202 impact hammer (Hottinger Brüel & Kjær A/S, Virum, Denmark) and B&K type 4395 ICP accelerometers (Hottinger Brüel & Kjær A/S, Virum, Denmark). The measuring system was used an 8-channel vibration conditioner Sirius+ (DEWESoft d.o.o., Trbovlje, Slovenia) with a DEWESoft X3 system software (DEWESoft d.o.o., Trbovlje, Slovenia). The upper hull plate was marked with 114 measuring points to be excited using an impact hammer. The system response was recorded using seven accelerometers arranged in the configuration shown in Figure 2. In this diagram white and orange dots indicate where the hammer struck, while orange dots indicate accelerometer locations. The distribution of measurement points is dictated by two factors. The first is the reliable representation of the vibration modes. The denser the measurement grid, the greater the accuracy of the description of the successive modes of vibration. If the measurement grid is too sparse, there is a risk that the vibration modes will not be detected or will be misidentified [22]. The second factor in limiting the grid’s density are the areal or local stiffening of the structure, such as structural reinforcement profiles (Figure 2), elements allowing the installation of additional panels to increase the protection of the crew, and structural openings. The roof hull plate has two such openings. The first, located at the front, is intended for the installation of the turret system. The second is located in the rear section and is used to install hatches. Seven accelerometers were placed across the entire plate surface. Two at the level of the turret opening (Nos. 1 and 2), two near the hatch opening (Nos. 6 and 7) and three (Nos. 3, 4, 5) between the structural openings. The location of the accelerometers was determined based on preliminary numerical tests. Increased vibration amplitudes of the roof hull plate were observed for the indicated locations.

During measurements, the accelerometer positions remained fixed, while the hammer was moving. Hull vibrations were induced by striking successive structure points. At each measuring point, the impact force was assessed based on the exceedance of the minimum and maximum values and the occurrence of double impact phenomenon. To improve the quality of the results, three strokes were taken and averaged for each measuring point.

The Dewesoft System recorded and analysed the real-time input and output signals by calculating the transition function described by Equation (1) (in the analysis, the H1 estimator was used) [23]:

(1) $$TF(\omega )=\frac{S_{xy}(\omega )}{S_{xx}(\omega )}$$

where *S_xy_*(*ω*) is the cross spectral density in the frequency domain of input *X*(*t*) (force) and output *Y*(*t*) (acceleration) and *S_xx_*(*ω*) is the auto spectral density in the frequency domain of input *X*(*t*) (force).

During tests, an H1(*ω*) estimator was used, which assumes a noisy output signal when compared with the input signal. The function was defined for each excitation–response pair, resulting in 798 transition functions for the test object. The occurrence of resonance was indicated by reaching the maximum of the local transition function with a simultaneous phase change. To confirm this, the coherence function was also calculated, and the MIF value was calculated using relation (2) [24]:

(2) $$MIF=1-\left(\frac{\sum_{i,j=1}^{n}Real\left(TF_{ij}(\omega )\right)\cdot \left|TF_{ij}(\omega )\right|}{\sum_{i,j=1}^{n}{\left|TF_{ij}(\omega )\right|}^{2}}\right)$$

where *TF_ij_*(*ω*) is the transfer function between each excitation–response pair; *i* and *j* are the numbers of measured output (acceleration) and input (force) channels, respectively (*i* = 1, …, 7; *j* = 1, …, 114).

A MIF value close to 1 indicated the occurrence of another natural vibration frequency and its associated vibration mode shape. MIF is a vector channel calculated over all transfer functions (all points), meaning it is only one channel.

### 2.2. Numerical Study

**Figure 3:**
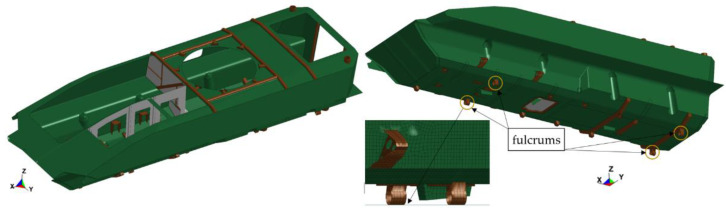
The vehicle hull model.

To determine the frequency and mode shape of natural vibrations, a numerical model of the transporter hull was prepared. The base hull model (Figure 3) is composed of approximately 147,000 fully integrated shell elements. The average edge length of the element is about 20 mm.

In addition to the hull plates themselves, the model takes into account the presence of structural reinforcements, the installation brackets, and structural and technological holes. These elements significantly affected the structure rigidity and, therefore, its vibration. The hull is supported at four points by one-sided ties (Rigidwall contact was applied). The whole structure is loaded with earth acceleration.

The hull of the vehicle is made of Armox 500T armour plate. The elastic–plastic material model with isotropic hardening including a strain rate effect was applied to describe the steel elements properties (Equation (3)) (the material data are presented in Table 1). The choice of the constitutive model of the material was dictated by the further intended use of the developed vehicle model. In future work, it is planned to carry out calculations of the strain on the structure caused by a rapidly changing load resulting from the recoil force of the cannon and the effect of a blast wave. The choice of the constitutive model was determined by the further purpose of the vehicle model. In the next stage of the work, it is planned to perform stress analysis of structure with quickly changing load resulting from the recoil force of cannon and impact of blast wave. The Johnson–Cook (JC) model provides a satisfactory prediction of flow stress for large strains and high strain rates when its dependence on strain rate is linear in a semi-logarithmic scale. This model is commonly used in modelling problems related to crash tests or the effects of explosive charges. This model is commonly used in modelling issues related to crash tests or blast loading [25,26,27]. The mathematical formula which describes this model is as follows [28,29]:

(3) $$\sigma \left(\varepsilon ,\dot{\varepsilon }\right)=\left(A+B\cdot \varepsilon^{n}\right)\cdot \left(1+C\cdot \ln\left(\frac{\dot{\varepsilon }}{{\dot{\varepsilon }}_{0}}\right)\right)$$

where *A* is the yield stress of the material under reference conditions, *B* is the strain hardening constant, *n* is the strain hardening coefficient, *C* is the coefficient responsible for the kinematic strengthening (for the strain intensity effects) and *ε*, $\dot{\varepsilon }$ describe the equivalent plastic strain and the equivalent plastic strain rate respectively and ${\dot{\varepsilon }}_{0}$ is the reference strain rate.

Mechanical properties and material data for Armox 500T are taken from results of experimental studies presented in paper [30]. During the numerical tests, in all configurations, the parameter values were not changed.

LS-Dyna software (Revision R12.0.0) (ANSYS, Inc., Canonsburg, PA, USA) was used to model the hull structure. Due to the large number of degrees of freedom for the considered structure, an iterative Lanczos algorithm designed to calculate the predetermined number of vibrational frequencies was used in the calculations. During the calculations, the 50 lowest natural vibration frequencies and associated vibration mode shapes were determined. This required solving a generalised eigenvalue problem system of equations which in matrix form could be written as Equation (4) [28]:

(4) $$(K-M\omega_{0}^{2})\cdot \Psi =0$$

where ***ω_o_*** is the natural frequency vector, Ψ is the mode shape vector, K is the stiffness matrix of the system, and M is the inertia matrix.

In terms of this work, the most important aspect was the vibration of the roof hull located above the combat and landing compartment. Due to the existing connections between the hull plates (top, side, and bottom), it was not possible to isolate the area under consideration (roof hull plate) for calculations. This procedure changed the boundary conditions at the plate edges, thereby affecting the calculated values of the natural vibration frequency and inducing changes in the vibration mode shape.

**Figure 4:**
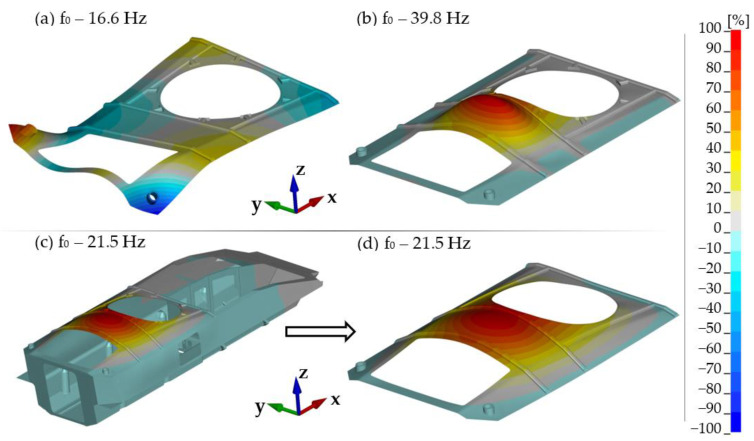
Natural vibrations for various boundary conditions: (**a**) free plate, (**b**) fixed on the edges, (**c**) base hull (Conf. 1), (**d**) view of roof hull plate (the same configuration as (**c**)).

Figure 4 presents an example illustrating the impact of boundary conditions on natural frequencies and mode shape of the roof hull plate. Calculations were made for a free plate (Figure 4a) and one fixed on its edge (Figure 4b). Figure 4c shows the calculation of the obtained results for the entire hull. For ease of comparison with the previous ones, Figure 4d shows only the roof fragment. 21.5 Hz is the lowest natural frequency associated with the roof hull plate only. Of course, there are also lower ones, but they are associated with the natural vibrations of the hull as a whole (mainly bending and torsion of the hull in various planes).

The results obtained for the free plate significantly underestimate the natural frequency (16.6 Hz), while the introduction of fixed boundary conditions increases the stiffness of the system and thus overestimates the natural frequency (39.8 Hz). In order to correctly map its working conditions, it is necessary to include a whole hull in the calculations.

### 2.3. Hull Configurations

**Figure 5:**
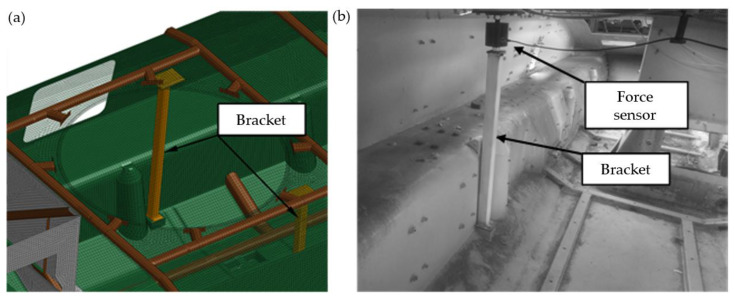
Conf. 2 (base hull and two roof hull plate brackets): (**a**) FEM model, (**b**) test object.

**Figure 6:**
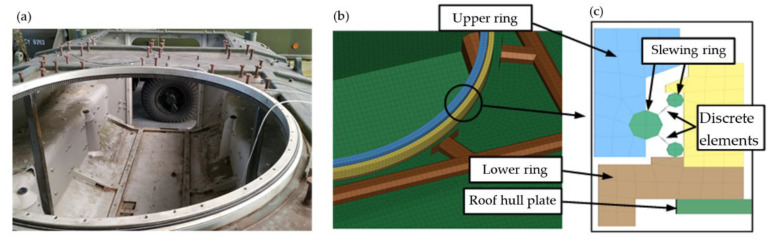
Conf. 3 (base hull, two roof hull plate brackets and bearing): (**a**) test object, (**b**) FEM model, (**c**) FEM model cross section.

**Figure 7:**
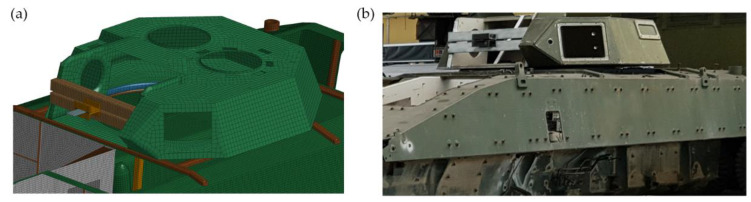
Conf. 4 (base hull, two roof hull plate brackets, bearing and turret): (**a**) FEM model, (**b**) test object.

The following hull configurations (Conf.) were considered during experimental and numerical studies:

Conf. 1. Base hull (Figure 3);
Conf. 2. Conf. 1 + two roof hull plate brackets (Figure 5);
Conf. 3. Conf. 2 + a turret bearing (Figure 6);
Conf. 4. Conf. 3 + a turret (Figure 7).

Supports were installed inside the transporter to reduce the load on the roof plate. During experimental studies, their design was modified to include the installation of force sensors. This made it possible to directly measure the forces transmitted from the plate under the turret to the bottom of the hull.

An important unit is the turret bearing. It enables the turret to rotate and is responsible for transferring forces between the hull and the transporter turret. Not including it in the model significantly alters the response of the structure to the applied loads. Preliminary investigations showed that the introduction of rigid connections between the turret and the hull (e.g., at the locations of the rolling rollers) did not make it possible to obtain the plot courses recorded in the experimental studies.

In the literature it is possible to find proposals for different solutions to the bearing modelling issue. They are largely related to load tests at the point of contact between the rolling elements and the rails [31,32], or tests of motion resistance [33]. To develop the model, solid elements are mostly used, while flexible models are used to describe material properties. Bearings were considered as an immediate item of interest in the referenced publications. They were not a part of a more complex load-bearing rotating structure. For applications where the bearing is one of many components, certain simplifications are usually made. In the papers [34,35,36,37,38], substitute models of the bearing balls were used. They decided to use the truss element and a spring with non-linear characteristics.

In the case of the object under consideration, modelling a full bearing with rollers would complicate and increase calculation time. The bearing rails were modelled with solid elements, while the rolling elements were represented by elastic-damping elements with non-linear characteristics. This made it possible to take into account the elasticity of the entire bearing and the internal clearance present.

### 2.4. Methodology of Comparison of Vibration Mode Shapes

***MAC*** was used to compare and determine differences between experimental and model studies of natural vibrations. The ***MAC*** value was determined using Equation (5) [39]:

(5) $$MAC(i,j)=\frac{{\left|{\left\{\Psi_{e}\right\}}_{i}^{T}{\left\{\Psi_{s}\right\}}_{j}\right|}^{2}}{\left({\left\{\Psi_{e}\right\}}_{i}^{T}{\left\{\Psi_{e}\right\}}_{i})({\left\{\Psi_{s}\right\}}_{j}^{T}{\left\{\Psi_{s}\right\}}_{j}\right)}$$

where ***Ψ_e_*** is the mode shape vector from the experimental test and ***Ψ_s_*** is mode shape vector from FE analysis.

Using this criterion, the mode shape vector obtained from model studies can be compared with the one produced by the experimental studies. A value close to 0 indicates no similarity, while a value of 1 means the two compared vectors are identical. In practice, two mode shape vectors were assumed to show significant correlation if the MAC value was greater than 0.9 while MAC values below 0.6 indicated no vector correlation.

**Figure 8:**
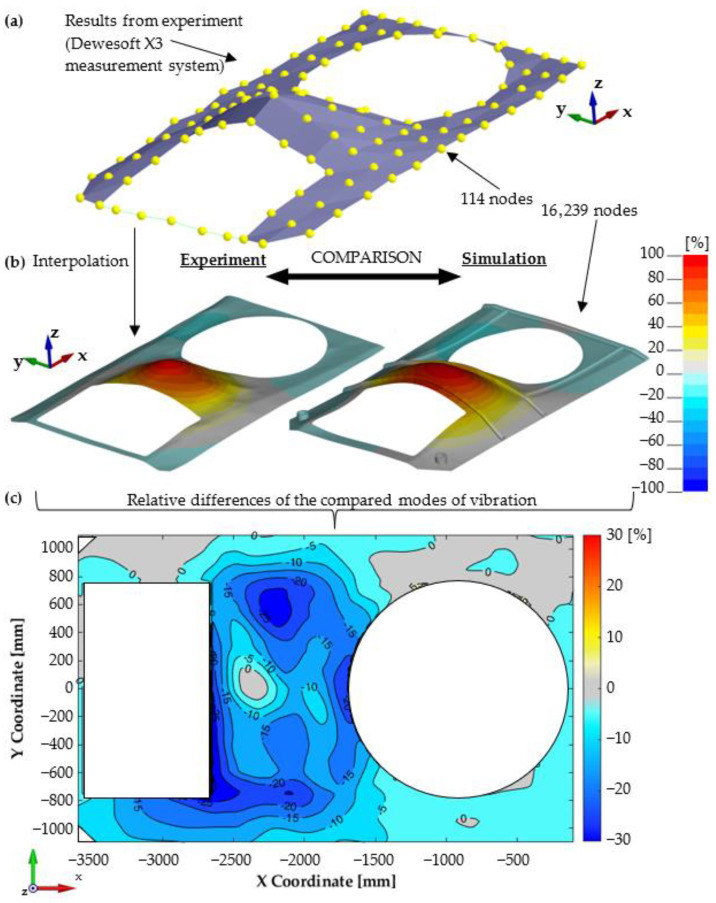
Vibration mode shape comparison: (**a**) Results from experiment (coarse point grid), (**b**) visual mode comparison (finer experimental grid after interpolation, equal to FE model), (**c**) relative difference of compared modes (experimental vs. numerical investigation).

The MAC criterion provides a quantitative comparison of the compatibility of the two mode shapes, but does not provide information regarding location differences on the investigated plate surfaces. To narrow down the areas with differences in the obtained natural vibration mode shapes, the differences between the vertical displacements of the individual nodes from experimental and numerical studies were calculated. Both mode shape vectors were normalised prior to comparison. The basic difficulty was the different number of analysed nodes between the experimental and numerical studies (114 and 16,239, respectively). Therefore, the mesh of the experimental results was modified to match the mesh used for numerical calculations. The values for additional points were determined using the cubic interpolation procedure, a new approach not found in the literature. Figure 8 shows a graphical representation of the experimental procedure. The starting point for the comparison was determined using the Dewesoft X3 system (DEWESoft d.o.o., Trbovlje, Slovenia) results for a coarse measuring grid. On this basis, using the interpolation procedure, an accurate grid was generated with the density used for numerical calculations. Finally, the distribution of the relative differences between vertical displacements for the entire plate area was obtained.

## 3. Results

### 3.1. Modal Analysis

Frequency values and natural vibration mode shapes were determined for various hull configurations. For each configuration, the four lowest frequencies were used for further comparison. For higher vibration frequencies, strong coupling between the roof hull plate and hull side plates was observed, and the obtained results were inconclusive.

**Figure 9:**
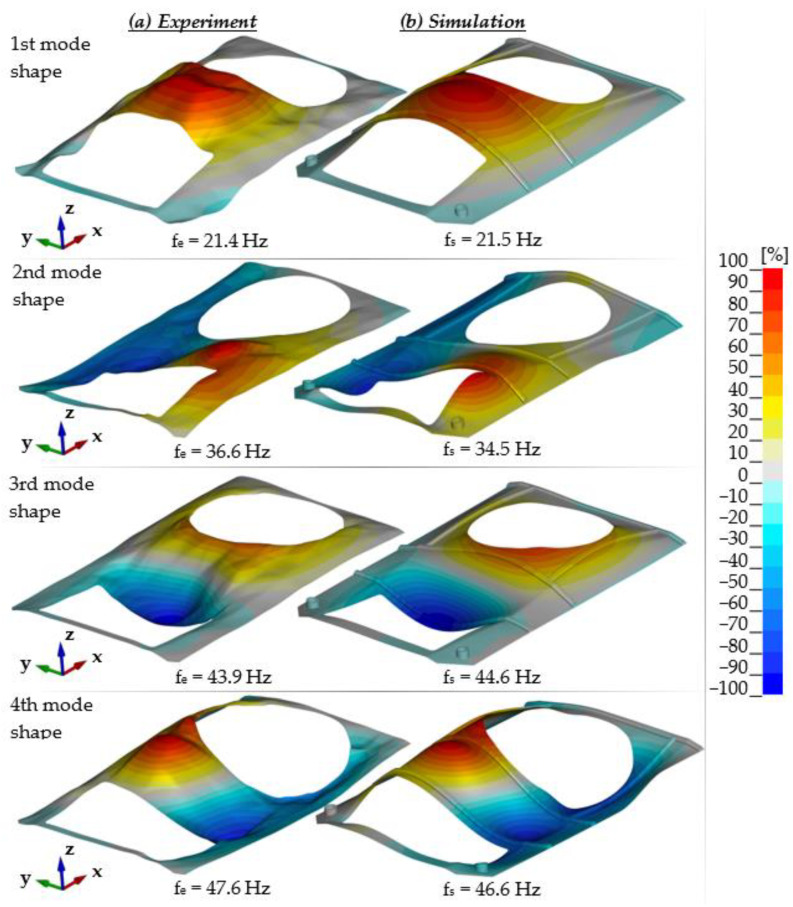
Frequencies and mode shapes of hull vibration.

**Figure 10:**
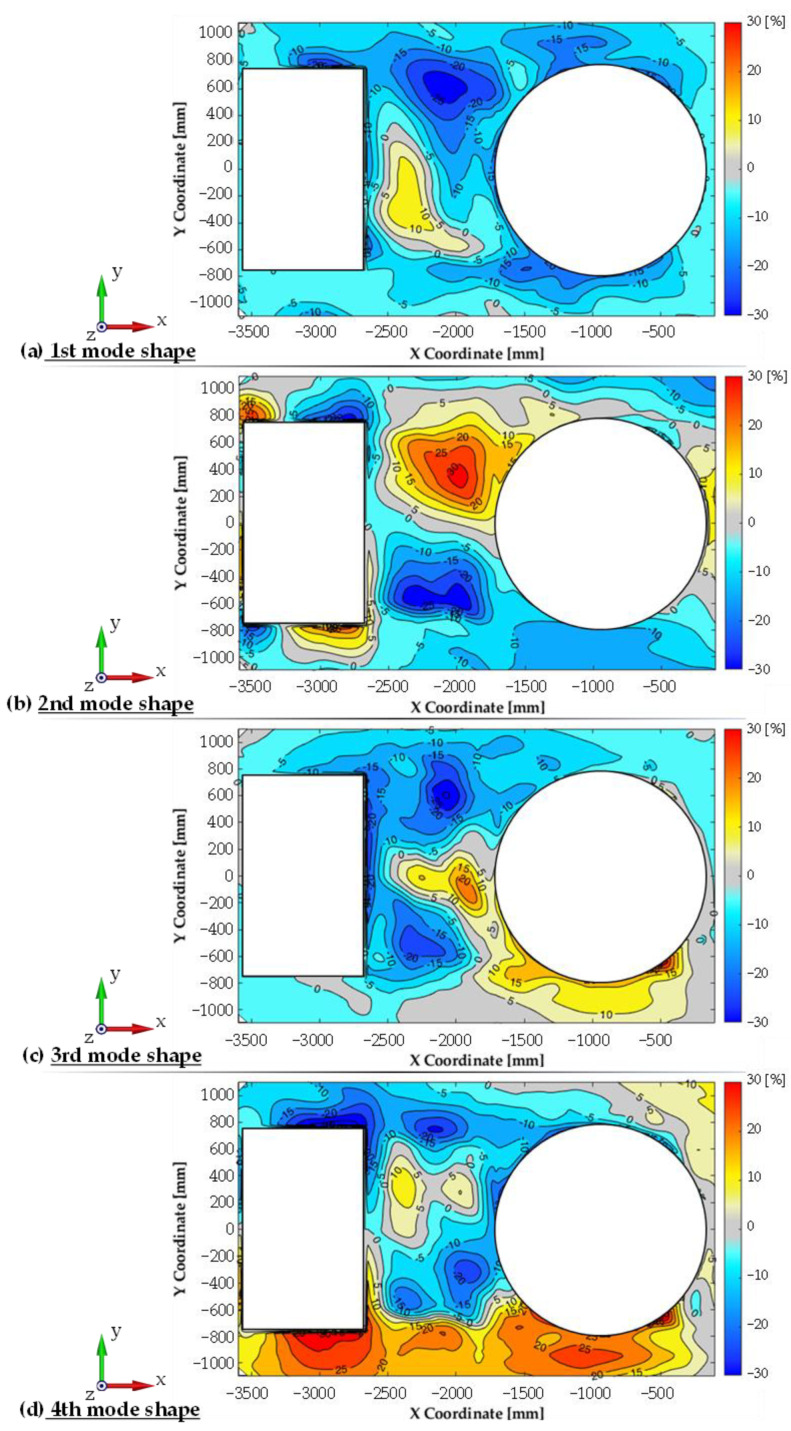
Relative differences in vibration mode shapes (real object vs. simulation).

For each configuration measurements were conducted using the modal hammer method for the adopted measuring grid. A similar modal analysis was performed using numerical studies. Figure 9 summarises the experimental and numerical test results for the first configuration, while Figure 10 shows a comparison of the relative differences for the first four mode shapes of transporter hull vibration. There is a high correspondence between the results, both in terms of the determined natural vibration frequency and form. In the majority of the plate, the differences in relative displacement did not exceed 10%. In a few small areas, larger differences were measured, but did not exceed 30%. At the same time, an increase in non-compliance for higher vibration mode shapes was observed.

**Figure 11:**
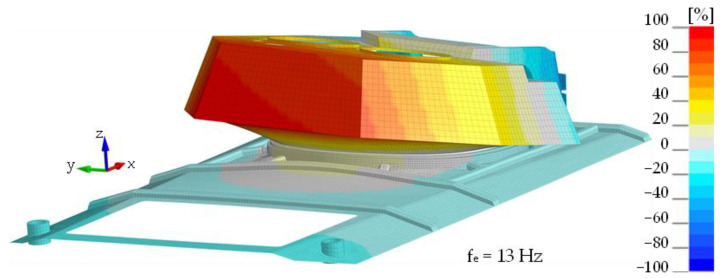
The first mode of vibration for the Conf. 4.- simulation.

**Figure 12:**
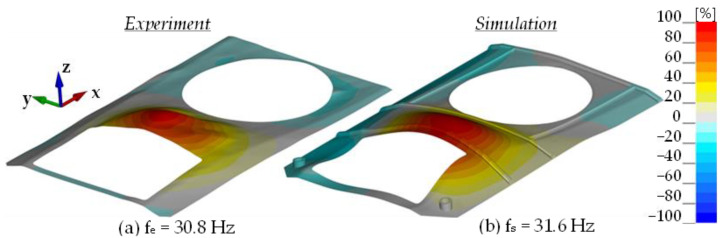
Second mode of vibration for the Conf. 4.

**Figure 13:**
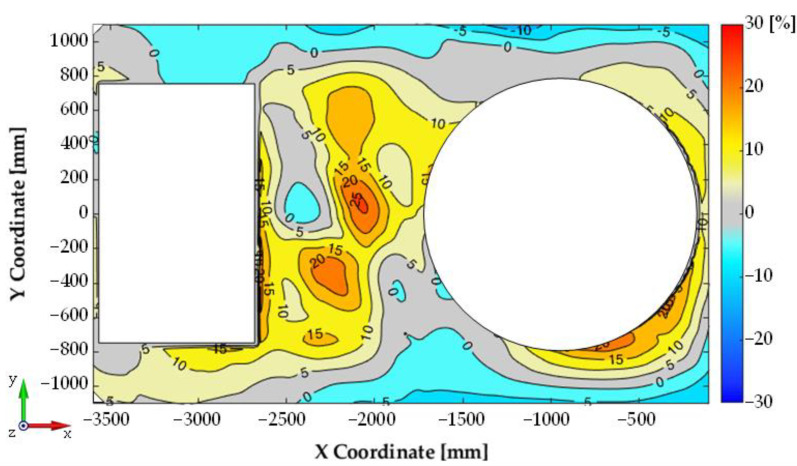
Relative differences in vibration mode shapes—Conf. 4.

The same figures for subsequent vibration frequencies of the plate were obtained using the mounted turret bearing and the roof hull plate brackets. Minor changes were observed in the frequency values associated with structural rigidity, and significant differences were seen in the turret-mounted configuration (IV). Using an impact hammer, it was not possible to generate vibrations associated with turret movement. Figure 11 shows the 1st shape obtained through FEM analysis is associated with rotational movement of the turret along the *y* axis. Figure 12 shows comparison of the experimental and numerical calculation results regarding the roof hull plate, and Figure 13 provides a map of relative differences in vibrational frequencies (experiment vs. simulation). In numerical calculations, the first frequency of plate vibration with the turret was approximately 13 Hz.

A detailed summary of the results obtained for all considered options is presented in Table 2.

### 3.2. Supplementary Experimental Test

**Figure 14:**
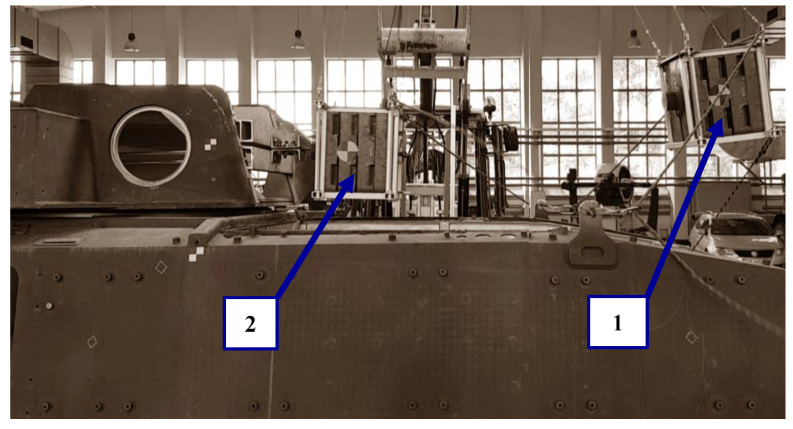
View of the hull during the test, where: “1”—initial position of a pendulum, “2”—position of pendulum before impacting the turret.

During the modal hammer tests, it was impossible to excite the turret-mounted hull structure in the lowest frequency range. As a result, an experiment was carried out, where the turret was hit with a pendulum (a moving rigid body with a mass of 350.0 kg presented in Figure 14). To avoid abrupt deceleration of the solid object, a friction dampener was installed on the turret which, by changing the pre-tension force, made it possible to change the intensity and duration of the force pulse. The kinetic energy of the body and deceleration time were modified to obtain a force pulse corresponding to the recoil resistance force when firing a 25.0–30.0 mm cannon. A force pulse of 770.0 N·s was obtained.

**Figure 15:**
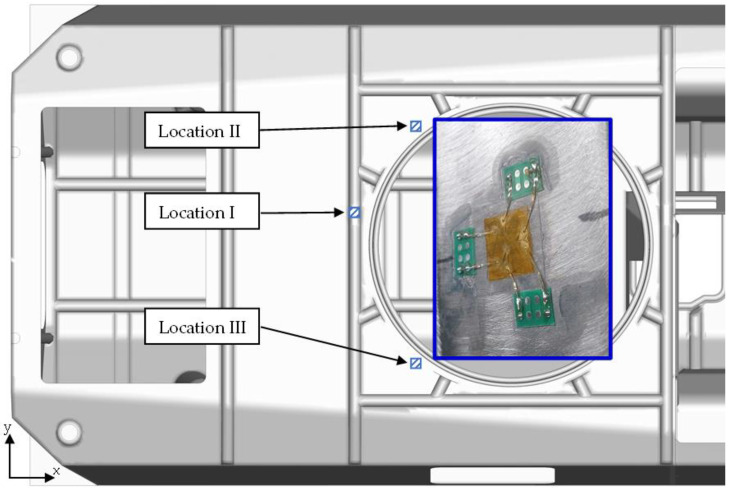
Distribution of strain gauge rosettes.

During the tests, the values of forces in the supports, the acceleration values at selected points of the plate and the deformation of the plate were measured. The arrangement of strain gauge rosettes is shown in Figure 15. Their location was selected on the basis of the results of preliminary simulation tests [26] indicating the areas of stress concentration on the roof hull plate that appear during firing. The first area is the reinforcement of the top plate of the transporter, located behind the turret opening in the plane of symmetry of the transporter. Unfortunately, due to the structural opening in the vicinity of this area, it was decided to move it away (Location I). The second and third areas are located between the brackets of the top plate supports and the bearing rail—on both sides. Due to the nature of the deformation of the plate, associated with its flexural vibrations, the dominant direction of deformation was the Y direction.

**Figure 16:**
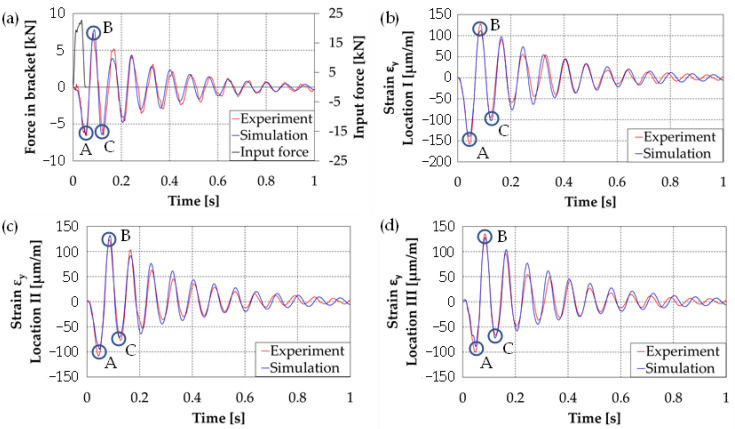
Comparison of results obtained from experimental and model studies: A, B, C-values of three successive amplitudes, (**a**) Force in bracket, (**b**) Strain ε_Y_ in location I, (**c**) Strain ε_Y_ in location II, (**d**) Strain ε_Y_ in location III.

Similar studies were repeated for the numerical model by introducing a force impulse. Figure 16 shows a comparison of the results: the axial force in the roof hull plate brackets and the strain courses of the plate at three points. The presented strains are with respect to the transverse direction (the longitudinal axis of the vehicle) for which the strain and stress values of reach their highest values.

In order to quantitatively compare the experimental and numerical test results, the MAC index and the correlation coefficient *R* [40] Equation (6) between the two data sets were calculated. Their values are summarised in Table 3.

(6) $$R(E,S)=\frac{1}{N-1}\sum_{i=1}^{N}\left(\frac{E_{i}-\mu_{E}}{\sigma_{E}}\right)\left(\frac{S_{i}-\mu_{S}}{\sigma_{S}}\right)$$

where, *S_i_* are data samples from experiment and simulation (FEM research), *µ_E,S_* are mean values for experimental and FEM datasets, *σ_E,S_* are standard deviations for experimental and simulation datasets, *N* is sample size.

The presented results show high consistency in terms of both vibration frequency and signal value. However, some differences were observed in the way the strain decayed. The experimental results showed a sharp decrease in strain values after two vibration cycles, while the numerical results are characterised by the disappearance of vibrations due to viscous attenuation. For the analysed points, the experimental strain values for the first inclination were about 8–13% higher than the calculated results.

Achieving high concordance results was possible thanks to accurate mapping of the hull structure, measurement of mass data, as well as taking into account the hull-tower connection through the bearing. Its simplified representation by means of rigid elements (lack of consideration of elasticity and clearances), makes it impossible to obtain the correct answer of the structure in numerical tests.

## 4. Summary and Conclusions

As a result of modal analysis, the natural vibration frequencies and mode shapes of a roof hull plate of an armoured personnel carrier hull, a hull extended by two cantilevers of the roof hull plate, and by a bearing and a turret were determined.

Through calculation and analysis, some conclusions were obtained:

1. The experimental test results for subsequent stages of construction completion were compared with the model test results and showed a good agreement in regards to both the vibration and frequency mode shapes. For the configurations considered, the relative frequency difference between experimental and model test results in most cases did not exceed 3%, reaching 6.1% in the worst case. For virtually all vibration mode shapes, the MAC index reached values above 0.9. The only exception was the fourth shape in the configuration with a fixed turret, where the MAC index reached the value of 0.86.
2. Modal analysis based on a roving hammer showed limitations related to the excitation of a complex structure with significant mass. Due to the insufficient magnitude of the transmitted force pulse as a result of a single impact, the model analysis did not show natural vibrations with a frequency of approximately 13 Hz, a value revealed by the model tests for the variant including a hull with brackets, a bearing, and a turret. This figure was associated with the turret movement and vibrations of its bottom plate.
3. Additional tests, including the pulsed load of the turret with a moving mass, showed a vibration frequency of approximately 13 Hz (both in the recorded strain at the three measuring points as well as the force in the brackets). A comparison of the results obtained by experimental and model studies allowed us to conclude that the developed numerical model largely reflects the construction of a real object.
4. Satisfactory validation of the model entitles the authors to conduct further research. The developed model will be used to determine the stress state of hull during on-board weapon firing as well as to assess what other weapon systems can be installed on this type of structure. In addition, it is also planned to complement the experimental and numerical studies with theoretical analyses, including the issues of natural vibrations and internal ballistics.

## Figures and Tables

**Table 1:** Mechanical Properties and Material Data for Armox 500T [30].

R_p0.2_ (MPa)	R_m_ (MPa)	A_5_ (%)	Hardness HBW	Elastic Modulus E (GPa)
1250 (minimum)	1450–1750	8 (minimum)	480–540	207
**A [MPa]**	**B [MPa]**	**n**	**C**	**m**
849	1340	0.0923	0.00541	0.870

**Table 2:** Comparison of Natural Vibration Frequencies from Experimental and Numerical Studies.

Hull Configuration	Frequency (Hz)		Natural Frequency Difference (%)	MAC
	Experiment	Simulation		
1	f_e1_ = 21.4	f_s1_ = 21.5	0.47	0.960
	f_e2_ = 36.6	f_s2_ = 34.5	6.1	0.960
	f_e3_ = 43.9	f_s3_ = 44.6	1.6	0.937
	f_e4_ = 47.6	f_s4_ = 46.6	2.1	0.924
2	f_e1_ = 30.5	f_s1_ = 31.4	3.0	0.944
	f_e2_ = 46.4	f_s2_ = 47.4	2.2	0.932
	f_e3_ = 53.7	f_s3_ = 51.9	3.5	0.985
3	f_e1_ = 31.1	f_s1_ = 31.7	1.9	0.944
	f_e2_ = 46.1	f_s2_ = 46.8	1.5	0.929
	f_e3_ = 52.2	f_s3_ = 49.7	5.0	0.939
4	f_e1_ = -	f_s1_ = 13.1	-	-
	f_e2_ = 30.8	f_s2_ = 32.1	4.2	0.961
	f_e3_ = 40.6	f_s3_ = 40.6	0.0	0.906
	f_e4_ = 56.8	f_s4_ = 56.8	0.0	0.860

**Table 3:** Summary of Experimental and Numerical Results.

Indicator		Force	Strain *ε_Y_*		
			Location I	Location II	Location III
MAC		0.889	0.902	0.885	0.892
R		0.944	0.950	0.941	0.944
Relative error (%)	A	3.95	11.6	12.0	11.6
	B	4.98	12.9	7.67	6.19
	C	2.75	7.41	6.74	8.10

## Data Availability

Not applicable.
